# Koalas vaccinated against Koala retrovirus respond by producing increased levels of interferon-gamma

**DOI:** 10.1186/s12985-020-01442-7

**Published:** 2020-10-31

**Authors:** Olusola Olagoke, Bonnie L. Quigley, Peter Timms

**Affiliations:** Genecology Research Centre, University of the Sunshine Coast, Sunshine Coast, QLD Australia

**Keywords:** Koala retrovirus, Vaccination, Immune response, mRNA, NanoString

## Abstract

Koala retrovirus (KoRV) is believed to be in an active state of endogenization into the koala genome. KoRV is present as both an endogenous and exogenous infection in all koalas in northern Australia. KoRV has been linked to koala pathologies including neoplasia and increased susceptibility to *Chlamydia*. A KoRV vaccine recently trialled in 10 northern koalas improved antibody response and reduced viral load. This communication reports the expression of key immune genes underlining the innate and adaptive immune response to vaccination in these northern koalas. The results showed that prior to vaccination, IL-8 was expressed at the highest levels, with at least 200-fold greater expression compared to other cytokines, while CD8 mRNA expression was significantly higher than CD4 mRNA expression level. Interferon-γ was up-regulated at both 4- and 8-weeks post-vaccination while IL-8 was down-regulated at 8-weeks post-vaccination.

## Background

KoRV is currently undergoing endogenization into the koala genome, thus offering an exciting opportunity to study retroviral endogenization in real time [1, 2]. Nine KoRV subtypes (KoRV-A to -I) have been identified with the subtypes A and B being the most studied [3]. All northern koalas (Queensland and New South Wales) are thought to be harbour at least KoRV-A, a KoRV subtype which is both endogenous and infectious in these koala populations. KoRV infection has been linked to immune alterations, with infected southern koalas shown to have altered immune profiles [4] and KoRV-B infected northern koalas shown to possess levels of immune dysregulation [5]. KoRV has also been linked to increased susceptibility to diseases in koalas [6]. We recently described antibody production and reduction in viral load following vaccination in koalas harbouring endogenous KoRV [7]. Briefly, koalas were vaccinated with a recombinant KoRV Env protein along with a Tri-adjuvant of poly I:C, polyphosphazine and host defence peptide 1002. Each animal received the vaccine subcutaneously on day zero with a booster dose delivered four weeks after. The vaccine was shown to lead to a significant increase in serum anti-KoRV IgG levels and neutralising antibody titres. The antibodies were shown to be cross-reactive against exogenous KoRV subtypes. In the present study, we examined the expression of important koala cytokines, immune markers and host restrictions factors to determine their pre- and post-vaccination levels in northern koalas harbouring endogenous KoRV.

## Main text

To understand the expression profiles of key immune genes associated with vaccine response, we compared the pre-vaccination levels to 4- and 8-weeks post-vaccination levels. We chose to analyse the gene expression changes in a non-antigen stimulated model. Genes targeted included nine cytokines (CCL4L, Interleukin (IL)-1β, IL-4, IL-6, IL-8, IL-10, IL-17A, IL-18 and Interferon gamma (IFN-γ), four host restriction factors (BST2, ISG15, RSAD2 and TRIM1) and two T-cell markers (CD4 and CD8β). Total RNA was purified from 200 µl whole koala blood using TRIzol® LS (ThermoFisher), as per manufacturer’s instructions. RNA was treated with the Turbo DNA-free kit (ThermoFisher) before target gene transcripts were quantified using a custom nanoString probe panel (Systems Biology and Data Science, Griffith University, Australia). Five koala housekeeping genes (ACTB, GAPDH, Hmg20a, Nckap1l and Stx12) were included in the panel for normalization and quality control. Normalized transcripts were quantified pre-vaccination and converted to fold-change for post-vaccination analysis.

**Fig. 1:**
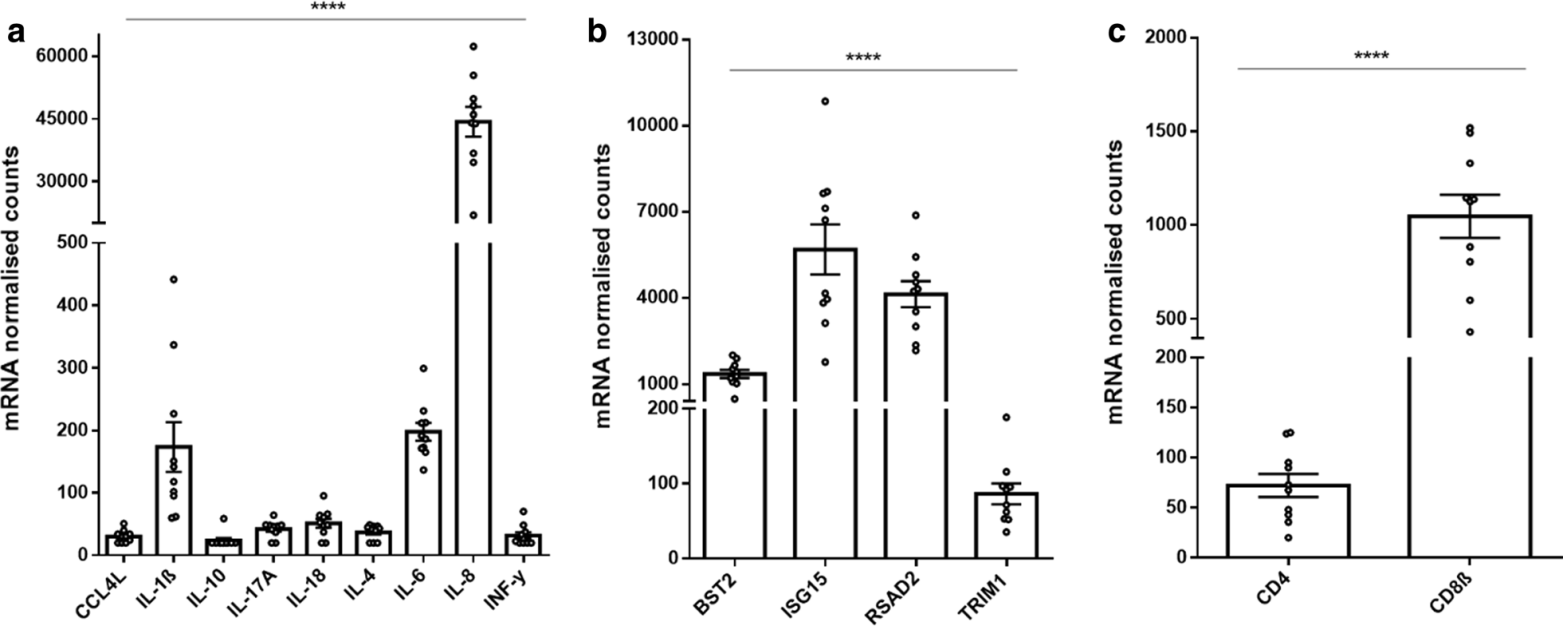
Baseline mRNA expression of key immune genes in koalas harbouring endogenous KoRV (n = 10). Data is presented as mean ± SEM. **a** Comparison of koala cytokine mRNA expression pre-vaccination. Analysis was done using one-way ANOVA (Fisher’s LSD). **b** comparison of the mean expression of host restriction factor genes in koalas (one-way ANOVA. Fisher’s LSD). **c** comparison of baseline CD4 and CD8 expression in koalas (Student’s T-test, *p* < 0.05). ****p < 0.0001

Expression levels of the target genes prior to vaccination were examined to gain an insight into the baseline immune genes expression of koalas harbouring endogenous KoRV. IL-8 was by far the most abundant cytokine mRNA expressed in these KoRV infected koalas (Fig. 1a), with over 200-fold higher expression compared to other cytokines. Both IL-6 and IL-1β were the next most abundantly expressed cytokines and they had comparable expression levels (Fig. 1a). The remaining cytokines tested (CCL4L, IL-10, IL-17A, IL-18, IL-4 and IFN-γ) had low, but detectable levels in all 10 koalas investigated (Fig. 1a). Additionally, all four host restriction factor genes (BST2, ISG15, RSAD2 and TRIM1) were expressed at quantifiable levels in all 10 koalas (Fig. 1b). Finally, CD4 gene expression levels were significantly lower than CD8β gene expression levels (~ 10 folds) (Fig. 1c).

**Fig. 2:**
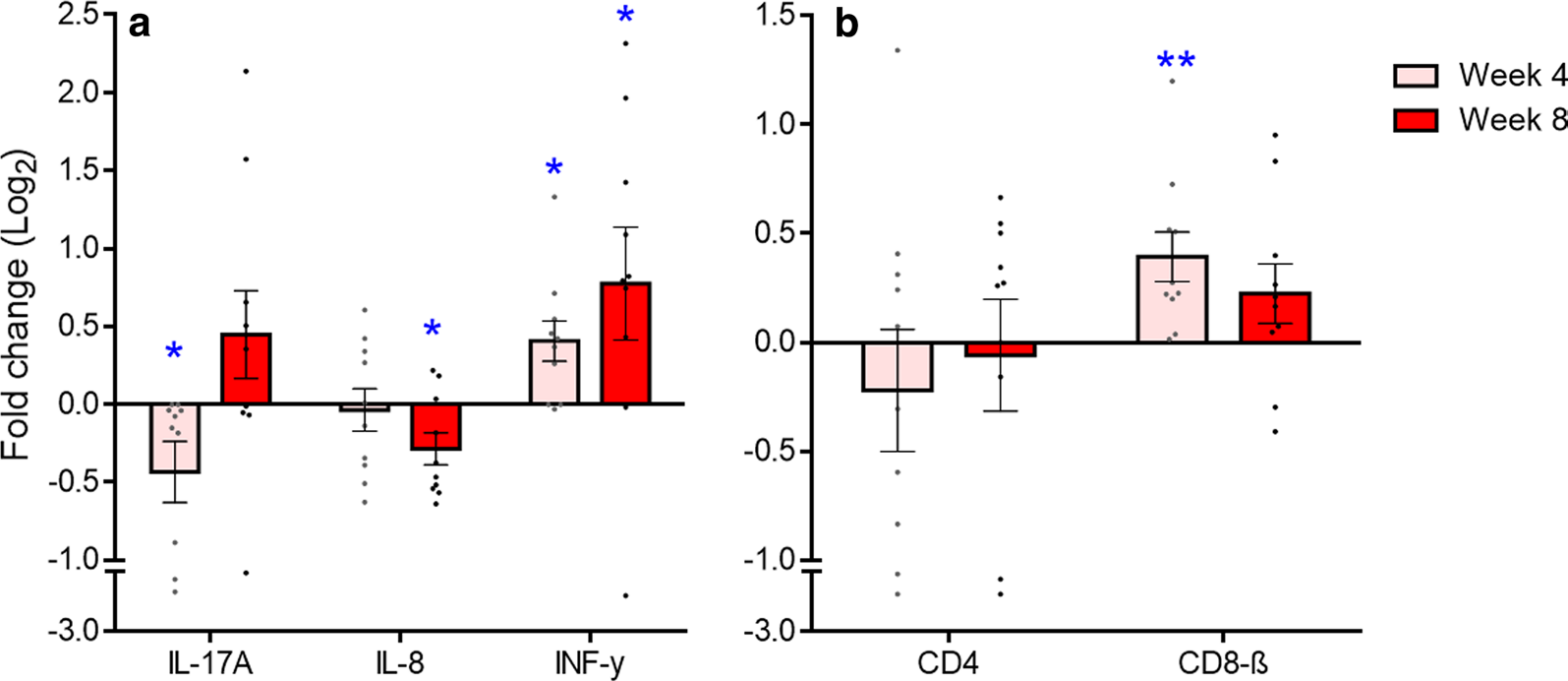
Fold change (Log_2_) in the mRNA expression of key immune genes in koalas vaccinated against KoRV (n = 10). Expression levels at 4- and 8-weeks post-vaccination were compared to pre-vaccination (Week 0) levels. Data is presented as mean ± SEM. **a** Comparison of koala cytokine mRNA expression. **b** comparison of CD4 and CD8 expression. The level of significance was measured using student’s T-test (*p* < 0.05). *p < 0.05, **p < 0.01

After vaccination, IFN-γ had the most pronounced expression change, with significant up-regulation at 4-weeks (Log_2_ fold change = 0.4; *p* = 0.006) and continued up-regulation at 8-weeks (Log_2_ Fold change = 0.8; *p* = 0.046) post-vaccination (Fig. 2a). There were also a small but statistically significant down-regulation of IL-17A at 4-weeks post-vaccination (Log_2_ Fold change = − 0.4; *p* = 0.039) and IL-8 by 8-weeks post-vaccination (Log_2_ Fold change = − 0.3; *p* = 0.012) (Fig. 2a). There were no significant changes in the mRNA expression of the other cytokines tested (CCL4L, IL-1β, IL-10, IL-18, IL-4, and IL-6) at either 4- or 8-weeks post-vaccination, data not shown. In addition, none of the host restriction factors tested (BST2, ISG15, RSAD2 and TRIM1) had significant changes in their expression either at 4- or 8-weeks post-vaccination when compared to pre-vaccination levels. Interestingly, the post-vaccination expression of CD4 mRNA remained largely unchanged relative to pre-vaccination levels, while CD8β mRNA was slightly upregulated at 4-weeks post-vaccination (Log_2_ Fold change = 0.4; *p* = 0.003) (Fig. 2b).

In this cohort of KoRV vaccinated and endogenously infected koalas, a small but significant increase in the expression of IFN-γ at both 4- and 8-weeks post-vaccination was observed, compared to pre-vaccination levels. While the biological impact of such sustained up-regulation is not fully known yet, a recent study showed that KoRV-positive southern (Victorian) koalas had significantly lowered IFN-γ levels compared to KoRV-negative koalas [4]. The authors of that study argued that such decreased IFN-γ levels may increase susceptibility to *Chlamydia* and other opportunistic infections in KoRV-positive koalas. This work is the first to report an increase in IFN-γ in response to KoRV vaccination and, as such, suggests a means to counteract KoRV-associated reduction in IFN-γ levels in koalas. The antiviral properties of IFN-γ have been well documented. For instance, long-lasting porcine circovirus vaccine efficacy was shown to be sustained, in part, by IFN-γ producing cells [8]. IFN-γ has also been linked to B-cell differentiation into IgG production [9]. The increase in IFN-γ detected in this work may offer insight into the sustained anti-KoRV IgG production and reduction in viral load reported in koalas following vaccination [7]. A possible mechanism for the observed increase in IFN-γ levels post-vaccination may be the inclusion of Poly[di(sodium carboxylatoethylphenoxy)phosphazene] (PCEP) as an adjuvant component in the KoRV vaccine. PCEP has been previously shown to induce a strong IFN-γ response and enhance antigen-specific immune response in mice following vaccination [10].

Several host restriction factors able to interfere with multiple stages of the viral life cycle have been identified in humans and animals [11]. Our study investigated the expression of four host restriction factors in koalas harbouring endogenous KoRV: BST2, ISG15, RSAD2 and TRIM1. All four genes were found to be expressed at quantifiable levels. Surprisingly, BST2, ISG15 and RSAD2 were expressed at relatively high levels, suggesting that these restriction factors may have biological importance in koalas harbouring endogenous KoRV. Although there was not a vaccine-induced up-regulation in these host restriction genes, the detection and investigation of these genes nonetheless present a new line of research into innate antiviral mechanisms for koalas.

The role of KoRV in modulating the CD4:CD8 ratio in koalas has been previously investigated. A study of captive koalas harbouring endogenous KoRV reported a CD4:CD8 expression median ratio of 2.1 (range: 0.1–6.3) [12]. Surprisingly, all koalas in the current study had very low levels of CD4 mRNA expression when compared to CD8β mRNA expression. This observation was not improved by vaccination. When compared to the normal range in humans (1.5–2.5) [13], the levels observed in this work suggest an abnormally low CD4 expression in koalas. Unfortunately, the lack of koala-specific reagents prevented further investigation into whether the reduced CD4 mRNA expression directly translated into fewer CD4 T cells. However, a positive association between CD4 molecule and CD4 mRNA expression has been previously shown in human studies [14]. As such, these results could suggest an ongoing KoRV-associated immunosuppression, possibly through the preferential loss of CD4 T cells in KoRV-infected koalas, similar to what is seen in cats infected with feline leukemia virus and feline immunodeficiency virus [15].

Finally, IL-8 was by far the most expressed cytokine detected in tested koalas. Increased IL-8 expression has been implicated in several pathologies including leukemia [16]. High levels of IL-8 is also associated with progression to disease in people infected with HIV-1, when compared to those who were able to maintain natural control of the infection [17, 18]. The extremely high level of IL-8 seen in these KoRV infected koalas is highly interesting, as it may be an important biomarker for underlying conditions that may be KoRV-associated. In these endogenously infected koalas, KoRV vaccination led to a small but significant decrease in IL-8 mRNA expression. A recent study showed that reduction in IL-8 levels was required for *Chlamydia* clearance in KoRV-infected koalas [19]. If further studies can conclusively link IL-8 to koala pathologies, then the role of IL-8 inhibitors, such as through vaccination, should be further explored.

## Conclusion

This work described increased IFN-γ mRNA expression following KoRV vaccination in koalas harbouring endogenous KoRV. A very high expression level of IL-8 mRNA prior to vaccination, followed by a slight but significant decrease post-vaccination, was also observed. Finally, this work provides new insight into possible mechanisms for KoRV-associated immunosuppression in KoRV infected koalas.

## Data Availability

The data that support the findings of this study are available from the authors on request.

## Abbreviations

KoRV: Koala retrovirus
IL: Interleukin
IFN: Interferon

## References

[1] Tarlinton RE, Meers J, Young PR (2006). Retroviral invasion of the koala genome. Nature.

[2] Hobbs M (2017). Long-read genome sequence assembly provides insight into ongoing retroviral invasion of the koala germline. Sci Rep.

[3] Quigley BL, Timms P (2020). Helping koalas battle disease—recent advances in Chlamydia and Koala Retrovirus (KoRV) disease understanding and treatment in koalas. FEMS Microbiol Rev.

[4] Maher IE (2019). Altered immune parameters associated with Koala Retrovirus (KoRV) and Chlamydial infection in free ranging Victorian koalas (*Phascolarctos cinereus*). Sci Rep.

[5] Maher IE, Higgins DP (2016). Altered immune cytokine expression associated with KoRV B Infection and season in captive koalas. PLoS ONE.

[6] Xu WQ (2013). An exogenous retrovirus isolated from koalas with malignant neoplasias in a US Zoo. Proc Natl Acad Sci USA.

[7] Olagoke O (2020). Therapeutic vaccination of koalas harbouring endogenous Koala retrovirus (KoRV) improves antibody responses and reduces circulating viral load. NPJ Vaccines.

[8] Ferrari L (2014). Memory T cell proliferative responses and IFN-γ productivity sustain long-lasting efficacy of a Cap-based PCV2 vaccine upon PCV2 natural infection and associated disease. Vet Res.

[9] Vazquez MI, Catalan-Dibene J, Zlotnik A (2015). B cells responses and cytokine production are regulated by their immune microenvironment. Cytokine.

[10] Mutwiri G (2007). Poly[di(sodium carboxylatoethylphenoxy)phosphazene] (PCEP) is a potent enhancer of mixed Th1/Th2 immune responses in mice immunized with influenza virus antigens. Vaccine.

[11] Colomer-Lluch M (2018). Restriction factors: from intrinsic viral restriction to shaping cellular immunity against HIV-1. Front Immunol.

[12] Maher IE (2014). Expression profiles of the immune genes CD4, CD8β, IFNγ, IL-4, IL-6 and IL-10 in mitogen-stimulated koala lymphocytes (*Phascolarctos cinereus*) by qRT-PCR. PeerJ.

[13] McBride JA, Striker R (2017). Imbalance in the game of T cells: what can the CD4/CD8 T-cell ratio tell us about HIV and health?. PLoSPathog.

[14] Yu Y (2002). Correlation between the expression of CD4 and the level of CD4 mRNA in human B-cell lines. Cell Immunol.

[15] Hartmann K (2012). Clinical aspects of feline retroviruses: a review. Viruses.

[16] Wierda WG (2003). Plasma interleukin 8 level predicts for survival in chronic lymphocytic leukaemia. Br J Haematol.

[17] Dos Santos JS (2017). Host factor predictors in long-term nonprogressors HIV-1 infected with distinct viral clades. Curr HIV Res.

[18] Pananghat AN (2016). IL-8 alterations in HIV-1 infected children with disease progression. Medicine (Baltimore).

[19] Phillips S (2019). Antibiotic treatment of Chlamydia-induced cystitis in the koala is linked to expression of key inflammatory genes in reactive oxygen pathways. PLoS ONE.

